# Nocardia Bloodstream Infection: A Retrospective Clinical Analysis of Seven Cases in a Single Centre

**DOI:** 10.7759/cureus.8007

**Published:** 2020-05-07

**Authors:** Liling Liang, Ping Wang, Jiewei Cui, Zhixin Liang

**Affiliations:** 1 Department of Respiratory Medicine, First Medical Centre, Chinese People's Liberation Army General Hospital, Beijing, CHN

**Keywords:** nocardiosis, nocardia bacteremia, nocardia species, symptom, treatment, outcome

## Abstract

Objective: Nocardiosis is a rare opportunistic infection caused by the Nocardia species. Nocardia bacteremia is a life-threatening presentation of disseminated nocardiosis that presents diagnostic and therapeutic challenges. We performed this retrospective analysis in a Chinese hospital from 2010 to 2019 to describe the characteristics of this rare bloodstream infection.

Methods: We searched the database of the real-time nosocomial infection surveillance system and identified patients whose blood cultures showed Nocardia bacteria growth. The medical records of these patients were extracted and analyzed by two independent researchers. The data included age, gender, complicating disease, duration from blood drawing to reporting, clinical signs and symptoms, blood routine and C-reactive protein results, radiological examinations, sites of involvement, antibiotic treatments, and outcomes.

Results: Seven patients with Nocardia bacteremia were found. There were four male and three female patients, whose ages ranged from 41 to 75 years. Six (85.7%) patients had predisposing conditions and were administrated corticosteroids for various reasons before the identification of Nocardia infection. The most common symptom was fever (100%). Five patients presented with lung or skin involvement; meanwhile, three patients presented with brain involvement. One patient presented with pelvic and peritoneum involvement, respectively. The most common findings of chest CT imaging were consolidation, followed by nodules and cavitations. Trimethoprim/sulfamethoxazole was prescribed to all patients after the diagnosis of Nocardia bacteremia. Six patients recovered, and one patient ultimately died.

Conclusions: Nocardia bacteremia is a rare bloodstream infection that usually occurs in immunocompromised patients. Clinical manifestations of patients are nonspecific. It often causes multiple organ involvement, and early diagnosis and prompt aggressive interventions are important to improve the outcome of this disease.

## Introduction

Nocardiosis is a granulomatous disease caused by Nocardia species, aerobic environmental Gram-positive actinomycetes. It predominantly occurs in immunocompromised patients, including those with solid-organ or hematopoietic stem cell transplant, malignancies, human immunodeficiency virus infection, and those receiving long-term treatment with steroids or immunosuppressants [1-4]. Nocardia infection primarily manifests as pulmonary involvement because inhalation is the primary route of bacterial exposure. Extrapulmonary nocardiosis is also common and is characterized by abscess formation, which can occur in skin, pleura, peritoneum, pericardium, and mediastinum. The central nervous system (CNS) is the most common extrapulmonary location for nocardiosis. Nocardia bacteremia is less often encountered and has rarely been reported so far [5-7].

In this study, we retrospectively collected the detailed clinical data of all patients with Nocardia bacteremia in a large tertiary-level healthcare facility in Beijing, China, during a 10-year period. This study aims to share our clinical experience on this specific infection.

## Materials and methods

Study design and data collection

We retrospectively reviewed the clinical information of the patients with Nocardia spp. isolated from blood cultures from January 1, 2010, to December 31, 2019, at the First Medical Centre of Chinese People’s Liberation Army General Hospital (PLAGH, Beijing, China). It is one of the biggest comprehensive hospitals in China, with a 3000-bed capacity and 190,000 inpatients per year.

The patients were identified by searching the real-time nosocomial infection surveillance system, the database of the Department of Infection Management and Disease Control of the First Medical Centre of Chinese PLAGH. Patients’ data were collected from electronic medical records. We recorded the data including age, gender, complicating disease, duration from blood drawing to reporting, clinical signs and symptoms, blood routine and C-reactive protein results, radiological examinations, sites of involvement, antibiotic treatments, and outcomes. All records were analyzed by two independent reviewers.

Definitions

The diagnosis of Nocardia bacteremia was defined as isolation of Nocardia spp. from one or more blood cultures. The time of diagnosis was defined as the date when the blood culture positive result report was obtained. Disseminated Nocardia infection was defined as Nocardia spp. bloodstream infection or involvement of two or more noncontiguous sites in the setting of clinical and radiographic evidence of organ involvement (e.g., lung, skin, pleura, peritoneum, or brain lesions).

Nocardia species' identification

Blood culture and species identification was accomplished in the microbiology centre of PLAGH. Nocardia spp. identification was based on colony morphology on routine media and microscopic examination, and species were further identified using a battery of biochemical tests. 

Statistical analysis

Continuous variables were expressed as means, range, and standard deviations and categorical variables were expressed as frequencies and percentages. All statistical analyses were performed using the IBM Statistical Package for the Social Sciences (SPSS) for Windows, Version 22.0 (IBM Corp., Armonk, NY).

## Results

Characteristics of the patients

Through searching the real-time nosocomial infection surveillance system, we found seven patients with Nocardia bacteremia during the 10-year period in our centre. Characteristics of patients with Nocardia bacteremia are shown in Table 1.

**Table 1:** Demographic and clinical characteristics of patients with Nocardia bacteremia

Characteristics	Patients with Nocardia bacteremia (N=7) (n, %)
Patient demographics	
Age (years), mean ± SD (range)	55.6 ± 12.0 (41-75)
Male sex	4 (57.1)
Underlying conditions	
Primary nephrotic syndrome	2 (28.6)
Acute myelogenous leukemia	1 (14.3)
Thrombocytopenic purpura	1 (14.3)
Sarcoidosis	1 (14.3)
Pemphigus vulgaris	1 (14.3)
Cerebral infarction	1 (14.3)
Symptoms	
Fever	7 (100)
Chills	3 (42.9)
Cough/expectoration	5 (71.4)
Headache	3 (42.9)
Nausea/vomiting	1 (14.3)
Confusion	1 (14.3)
Seizure	1 (14.3)
Skin/soft tissue lesions	5 (71.4)
Septic shock	1 (14.3)
Laboratory data	
White blood cell (103 cells/mL)	10.39 ± 2.0 (7.65-13.64)
C-reactive protein, mg/dL	4.21 ± 1.8 (1.24-6.73)
Procalcitonin, ng/mL	0.32 ± 0.21 (0.05-0.68)
Sites of involvement	
Lung	5 (71.4)
Central nervous system	3 (42.9)
Skin/soft tissue	5 (71.4)
Other sites	2 (28.6)
Diagnostic-related information	
Days from admission to diagnosis of Nocardia bacteremia, mean ± SD (range)	15.7 ± 6.05 (7-25)
Days of blood culture report, mean ± SD (range)	7.43 ± 1.59 (5-10)
Polymicrobial bloodstream infection	1 (14.3)

**Figure 1:**
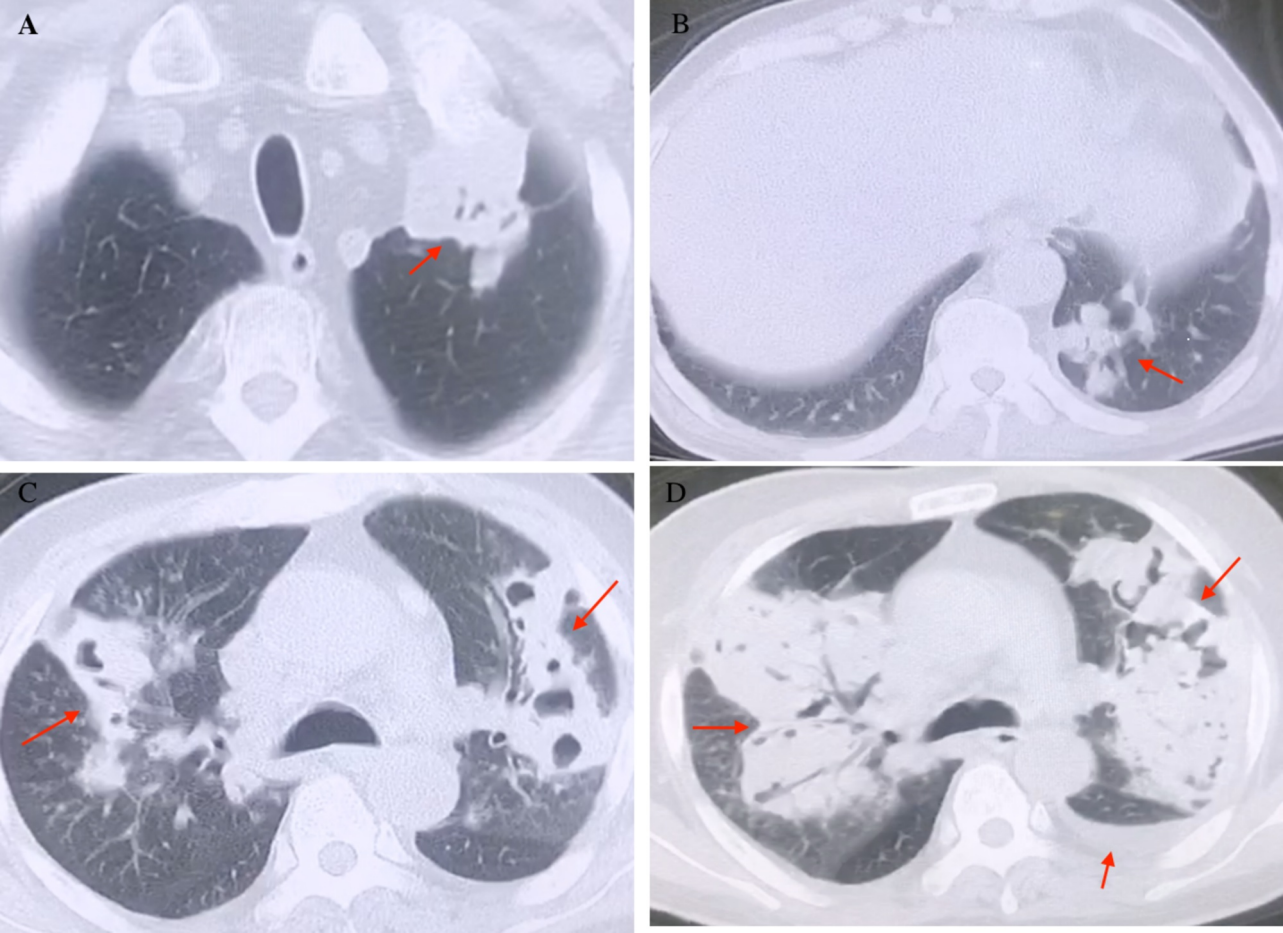
Chest CT of the patients. A: mass in the left upper lobe. B: multiple nodules in the left lower lobe. C: consolidation and multiple cavitations with air bronchogram in bilateral lobes. D: bilateral pulmonary diffuse lesions with pleural effusion

The age of patients ranged from 41 to 75 years. Four (57.1%) patients were male. Among the seven total patients, six (85.7%) were immunocompromised, receiving high-dose corticosteroid or immunosuppressant treatment due to underlying conditions. Accompanying diseases included primary nephrotic syndrome, acute myelogenous leukemia, thrombocytopenic purpura, sarcoidosis, and pemphigus vulgaris. One patient had a cerebral infarction. All patients were HIV-negative. Fever (100%) was the most common symptom in these patients. Other common symptoms included cough/expectoration (71.4%), chills (42.9%), and headache (42.9%). One (14.3%) patient experienced nausea/vomiting, confusion, seizure, and septic shock. The mean white blood cell count of patients was 10.39 ± 2.0 × 10^9^/L. The mean percentage of neutrophils was 75.35 ± 0.26. The mean C-reactive protein concentration was 4.21 ± 1.8 mg/dL, which was slightly beyond the normal range (0-0.8 mg/dL). The mean procalcitonin concentration was 0.32 ± 0.21 ng/mL, which was in the normal range (0-0.5 ng/mL). Five patients presented with lung or skin involvement, while three patients presented with brain involvement. The most common findings of chest CT imaging were consolidation (100%), followed by nodules (80%) and cavitations (80%). The CT imaging of one case presented as mass; pleural effusion was observed in another case (Figure 1).

**Figure 2:**
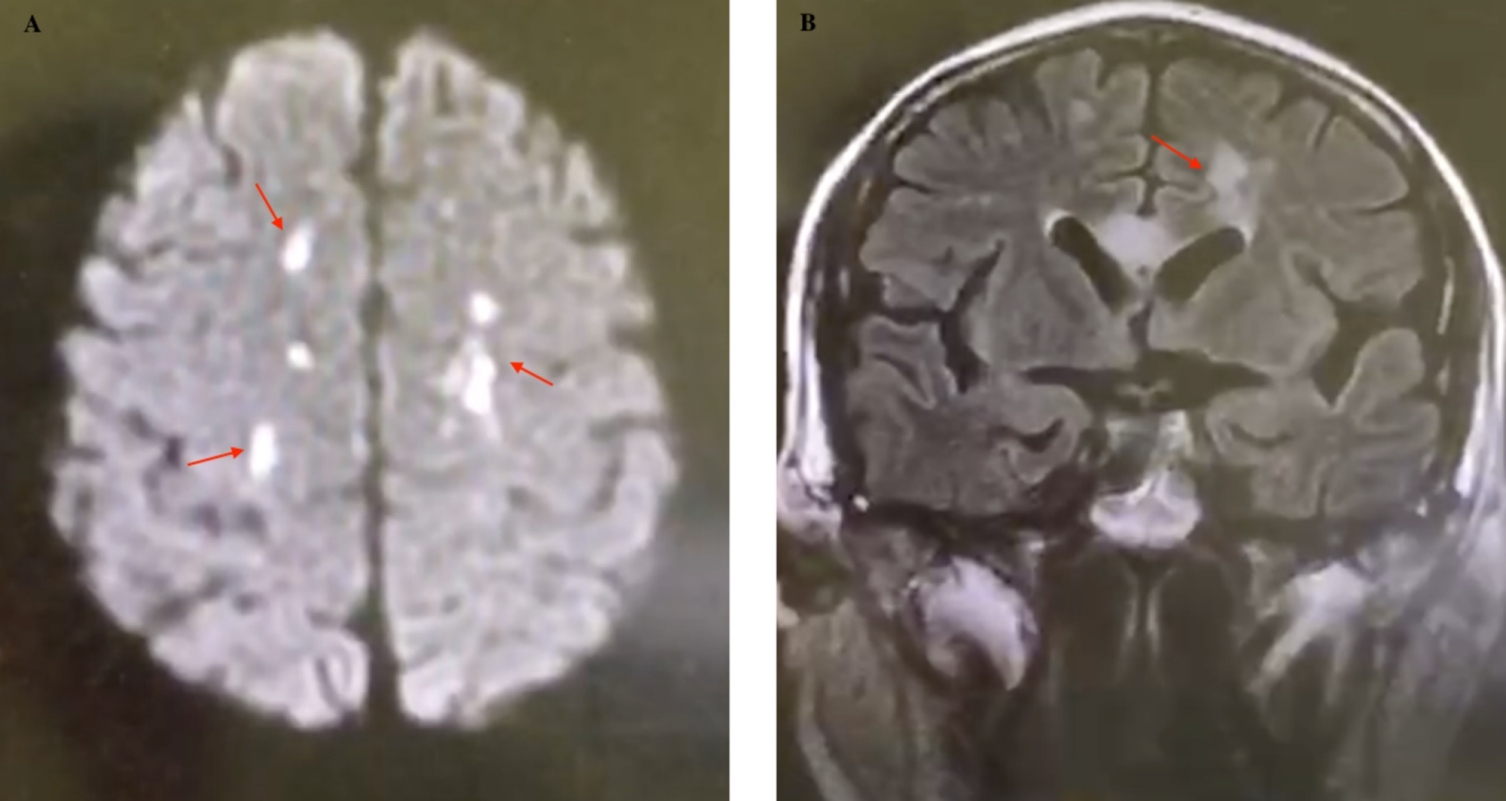
Brain MRI of patient 5 showed multiple focal lesions. A: multiple abnormal high signals in bilateral frontal lobes, semioval centre, and corpus callosum on DWI. B: multiple patchy and other slightly longer T1 and T2 signals in bilateral frontal lobes, semioval centres, and corpus callosum in the coronal image with no abnormal enhancement DWI, diffusion weighted image

Brain CT or MRI usually showed multiple abscesses (Figure 2). 

One patient presented with pelvic and peritoneum involvement. Polymicrobial bloodstream infection with Nocardia and Enterococcus faecium occurred in one (14.3%) patient. The interval of diagnosis was 15.7 ± 6.05 days, and the interval of blood culture report was 7.43 ± 1.59 days.

Nocardia species’ identification

Nocardia species were seen microscopically as beaded Gram-positive, thin, branching filamentous bacilli, with modified acid-fast stains. Seven isolates grew well on both blood agar and Löwenstein-Jensen media. 

Treatment and outcomes

Most patients were treated with empirical antibiotics for infections before positive culture of Nocardia. Trimethoprim/sulfamethoxazole (TMP/SMZ) was prescribed to all patients after diagnosis of Nocardia bacteremia. Two patients were administered only one medication, which was TMP/SMZ, while the other patients were treated with two or more antibiotics, including levofloxacin, meropenem, ceftriaxone, and linezolid. The daily therapeutic dose of TMP/SMZ ranged from 640 to 1280 mg of trimethoprim and 3200 to 6400 mg of sulfamethoxazole, and the mean duration of treatment was 13.6 ± 4.1 months (range, 5-18 months). Three patients had renal damage, and two patients had hepatic damage during treatment. The blood urea nitrogen, creatinine, glutamate-oxaloacetate transaminase, and glutamate-pyruvate transaminase levels of these patients remained stable after kidney and liver function-protecting treatment. The median follow-up period was 26.4 ± 10.4 months (range, 5-36 months). Six patients recovered, while one patient ultimately died, who was elderly and had been treated with steroids and ciclosporin A because of his primary nephrotic syndrome. He had disseminated nocardiosis with lung, peritoneum, and skin involvement. The patient also had coronary heart disease and had percutaneous coronary intervention stent implantation four years ago. He was intubated shortly after admission to the intensive care unit and died of multiple organ failure five months later. Details of patients with Nocardia bacteremia are shown in Table 2.

**Table 2:** Details of patients with Nocardia bacteremia ICU, intensive care unit; TMP/SMX, trimethoprim/sulfamethoxazole

Case number	Gender	Age (years)	Underlying disease	Associated risk factors	Medications	Other affected organ	Treatment	Outcome
1	Male	51	Pemphigus vulgaris	-	Steroids for 24 months	Lung + skin	TMP/SMX + levofloxacin	Recovered
2	Male	70	Cerebral infarction	-	-	-	TMP/SMX	Recovered
3	Female	44	Thrombocytopenic purpura	-	Steroids for 10 months	-	TMP/SMX	Recovered
4	Female	59	Sarcoidosis	-	Steroids for 8 months	Lung + brain + knee joint	TMP/SMX + levofloxacin	Recovered
5	Male	49	Acute myelogenous leukemia	-	Steroids for 14 months	Lung + brain + skin + pelvic	TMP/SMX + meropenem	Recovered
6	Male	75	Primary nephrotic syndrome	Coronary heart disease; endotracheal intubation; central vein catheterization; ICU stay	Steroids + cyclosporine for 8 months	Lung + skin + peritoneum	TMP/SMX + meropenem + levofloxacin	Died
7	Female	41	Primary nephrotic syndrome	-	Steroids + cyclosporine for 5 months	Lung + brain + skin	TMP/SMX + meropenem + levofloxacin + ceftriaxone + linezolid	Recovered

## Discussion

Nocardia is a Gram-positive bacterium that grows aerobically. It usually causes opportunistic infection especially in immunocompromised hosts, with the most common sites of infection being lung, brain, and skin. Nocardia bacteremia occurs less often and rarely has been reported. Here, we presented retrospectively the clinical characteristics of seven patients with Nocardia bacteremia. It is the first study of Nocardia bacteremia reported in China. 

As a rare pattern of nocardiosis, Nocardia bacteremia mostly developed in patients with depressed cell-mediated immunity. In our study, six (85.7%) patients had predisposing conditions and were treated with corticosteroids for various reasons before identification of Nocardia infection. It was noteworthy that one Nocardia bacteremia patient with cerebral infarction was apparently immunocompetent in this study. Recent studies showed that nocardiosis occurs in immunocompetent patients with increasing rates [4,8]. The underlying mechanism of susceptibility of this cohort remains unknown. It is worth mentioning that regardless of a patient’s immunologic status, the isolation of Nocardia from the respiratory tract or other body site should not be considered as a contaminating or colonizing organism. 

The symptoms of Nocardia bacteremia were usually nonspecific. They were sometimes confused with manifestations of primary disease, which increased the difficulty of diagnosis for clinicians. Fever was found in all seven patients with Nocardia bacteremia. The other common symptoms included cough/expectoration and chills. CNS is the most common extrapulmonary location for nocardiosis. The clinical manifestations of CNS involvement presented as headache, nausea/vomiting, confusion, and seizure. In this study, three (42.9%) patients with CNS involvement presented typical symptoms of headache, and one patient presented with nausea/vomiting, confusion, and seizure. Four (57.1%) patients presented with skin involvement and one presented with knee joint involvement. Extrapulmonary nocardiosis is characterized by abscess formation histopathologically. It represents a pyogenic bacterial process or develops into a chronic granulomatous lesion or mixed progressive inflammatory mass. 

Ninety-two Nocardia species have been reported to date, and 54 have been recognized as having clinical significance [9,10]. The most commonly isolated species in humans include N. asteroides, N. farcinica, N. cyriacigeorgica, N. nova, and N. brasiliensis [11-13]. No effective serological method has been developed for diagnosis of nocardiosis so far. A definitive diagnosis of nocardiosis is only made by the separation and identification of Nocardia species using the culture method that usually requires several days to several weeks [14,15]. A bacterial 16S rRNA sequence analysis has recently become the gold standard for the identification of Nocardia species [15]. It is with regret that it was not carried out in our centre, and we did not perform further identification of these species.

Nocardia bacteremia is an extremely severe form of disseminated nocardiosis with a higher mortality rate of approximately 60% [16]. Early diagnosis and treatment of Nocardia bacteremia are highly important to patients’ prognosis. TMP-SMZ is widely used as the mainstay drug in the treatment of nocardiosis [16-18]. Carbapenems, levofloxacin, amikacin, minocycline, ceftriaxone, and linezolid also have activity against Nocardia [10,17,18]. In the present study, TMP-SMZ was administered to each patient after the diagnosis of Nocardia bacteremia. Five patients received the combination of TMP-SMZ with carbapenems, levofloxacin, ceftriaxone, or linezolid. As for a disseminated refractory infectious disease, treatment of longer duration is needed for Nocardia bacteremia. The mean duration of treatment in our study was 13.6 ± 4.1 months. The common adverse reactions of high-dose TMP-SMZ include renal insufficiency, hepatotoxicity, and myelosuppression. In this study, discontinuations due to drug adverse reactions were not identified. TMP-SMZ is active against most Nocardia species; however, increasing resistance of Nocardia species to TMP-SMZ has been problematic, with resistance rates reported to be as high as 42% [19]. Combination therapy with TMP-SMZ and other alternative effective antimicrobial agents may provide enhanced activity while reducing the side effects of drugs. Therefore, combination therapy with quinolones, carbapenems, amikacin, or a third-generation cephalosporin and TMP-SMZ should be considered in severe cases [18,20]. In our study, six patients improved after treatment, and one patient ultimately died. We thought that this death was related to his multiple organ failure rather than just due to Nocardia infection.

## Conclusions

This study described the clinical presentations of Nocardia bacteremia in a tertiary hospital in Beijing, China. This rare infection usually occurs in immunocompromised patients. The diagnosis of Nocardia bacteremia is often delayed due to its nonspecific clinical manifestation and the absence of specific serological diagnostic methods of nocardiosis. The diagnosis of Nocardia bacteremia should be considered early in immunocompromised patients especially with multiple organ involvement. Prompt aggressive interventions are important to improve the outcome of patients. However, it is difficult to draw conclusions about the overall epidemiology and microbiology of Nocardia bacteremia due to the small sample size of the current study. Hence, studies involving larger sample sizes are needed to provide more information on Nocardia bacteremia.

## References

[1] Lebeaux D, Freund R, van Delden C (2017). Outcome and treatment of nocardiosis after solid organ transplantation: new insights from a European study. Clin Infect Dis.

[2] Shannon K, Pasikhova Y, Ibekweh Q, Ludlow S, Baluch A (2016). Nocardiosis following hematopoietic stem cell transplantation. Transpl Infect Dis.

[3] Wang HL, Seo YH, LaSala PR, Tarrand JJ, Han XY (2014). Nocardiosis in 132 patients with cancer: microbiological and clinical analyses. Am J Clin Pathol.

[4] Kim YK, Sung H, Jung J (2016). Impact of immune status on the clinical characteristics and treatment outcomes of nocardiosis. Diagn Microbiol Infect Dis.

[5] Lai CH, Chi CY, Chen HP, Lai CJ, Fung CP, Liu CY (2004). Port-A catheter-associated Nocardia bacteremia detected by gallium inflammation scan: a case report and literature review. Scand J Infect Dis.

[6] Kontoyiannis DP, Jacobson KL, Whimbey EE, Rolston KV, Raad II (2000). Central venous catheter-associated Nocardia bacteremia: an unusual manifestation of nocardiosis. Clin Infect Dis.

[7] Al Akhrass F, Hachem R, Mohamed JA (2011). Central venous catheter-associated Nocardia bacteremia in cancer patients. Emerg Infect Dis.

[8] Steinbrink J, Leavens J, Kauffman CA, Miceli MH (2018). Manifestations and outcomes of nocardia infections: comparison of immunocompromised and nonimmunocompromised adult patients. Medicine (Baltimore).

[9] Conville PS, Brown-Elliott BA, Smith T, Zelazny AM (2017). The complexities of nocardia taxonomy and identification. J Clin Microbiol.

[10] Fatahi-Bafghi M (2018). Nocardiosis from 1888 to 2017. Microb Pathog.

[11] Brown-Elliott BA, Brown JM, Conville PS, Wallace RJ Jr (2006). Clinical and laboratory features of the Nocardia spp. based on current molecular taxonomy. Clin Microbiol Rev.

[12] Larruskain J, Idigoras P, Marimón JM, Pérez-Trallero E (2011). Susceptibility of 186 Nocardia sp. isolates to 20 antimicrobial agents. Antimicrob Agents Chemother.

[13] Lai CC, Liu WL, Ko WC, Chen YH, Tan HR, Huang YT, Hsueh PR (2011). Multicenter study in Taiwan of the in vitro activities of nemonoxacin, tigecycline, doripenem, and other antimicrobial agents against clinical isolates of various Nocardia species. Antimicrob Agents Chemother.

[14] Tania CS, David HM, Jonathan RI, Sharon CC (2015). Nocardia species. In Mandell, Douglas, and Bennett’s Principles and Practice of Infectious Diseases 8th ed.

[15] Kurahara Y, Tachibana K, Tsuyuguchi K, Akira M, Suzuki K, Hayashi S (2014). Pulmonary nocardiosis: a clinical analysis of 59 cases. Respir Investig.

[16] Martínez Tomás R, Menéndez Villanueva R, Reyes Calzada S, Santos Durantez M, Vallés Tarazona JM, Modesto Alapont M, Gobernado Serrano M (2007). Pulmonary nocardiosis: risk factors and outcomes. Respirology.

[17] Imai K, Koibuchi T, Iwamoto A (2011). Pulmonary nocardiosis caused by Nocardia exalbida complicating Pneumocystis pneumonia in an HIV-infected patient. J Infect Chemother.

[18] Ono M, Kobayashi Y, Shibata T (2008). Nocardia exalbida brain abscess in a patient with follicular lymphoma. Int J Hematol.

[19] Uhde KB, Pathak S, McCullum I Jr (2010). Antimicrobial-resistant nocardia isolates, United States, 1995-2004. Clin Infect Dis.

[20] Li S, Song XY, Zhao YY, Xu K, Bi YL, Huang H, Xu ZJ (2015). Clinical analysis of pulmonary nocardiosis in patients with autoimmune disease. Medicine (Baltimore).

